# Constructing Endophenotypes of Complex Diseases Using Non-Negative Matrix Factorization and Adjusted Rand Index

**DOI:** 10.1371/journal.pone.0040996

**Published:** 2012-07-16

**Authors:** Hui-Min Wang, Ching-Lin Hsiao, Ai-Ru Hsieh, Ying-Chao Lin, Cathy S. J. Fann

**Affiliations:** 1 Institute of Public Health, Yang-Ming University, Taipei, Taiwan; 2 Institute of BioMedical Science, Academia Sinica, Nankang, Taipei, Taiwan; Queen’s University Belfast, United Kingdom

## Abstract

Complex diseases are typically caused by combinations of molecular disturbances that vary widely among different patients. Endophenotypes, a combination of genetic factors associated with a disease, offer a simplified approach to dissect complex trait by reducing genetic heterogeneity. Because molecular dissimilarities often exist between patients with indistinguishable disease symptoms, these unique molecular features may reflect pathogenic heterogeneity. To detect molecular dissimilarities among patients and reduce the complexity of high-dimension data, we have explored an endophenotype-identification analytical procedure that combines non-negative matrix factorization (NMF) and adjusted rand index (ARI), a measure of the similarity of two clusterings of a data set. To evaluate this procedure, we compared it with a commonly used method, principal component analysis with k-means clustering (PCA-K). A simulation study with gene expression dataset and genotype information was conducted to examine the performance of our procedure and PCA-K. The results showed that NMF mostly outperformed PCA-K. Additionally, we applied our endophenotype-identification analytical procedure to a publicly available dataset containing data derived from patients with late-onset Alzheimer’s disease (LOAD). NMF distilled information associated with 1,116 transcripts into three metagenes and three molecular subtypes (MS) for patients in the LOAD dataset: MS1 (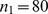), MS2 (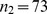), and MS3 (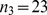). ARI was then used to determine the most representative transcripts for each metagene; 123, 89, and 71 metagene-specific transcripts were identified for MS1, MS2, and MS3, respectively. These metagene-specific transcripts were identified as the endophenotypes. Our results showed that 14, 38, 0, and 28 candidate susceptibility genes listed in AlzGene database were found by all patients, MS1, MS2, and MS3, respectively. Moreover, we found that MS2 might be a normal-like subtype. Our proposed procedure provides an alternative approach to investigate the pathogenic mechanism of disease and better understand the relationship between phenotype and genotype.

## Introduction

The identification of genes that contribute to human disease is an important step toward understanding disease etiology and can facilitate the development of diagnostic tools, preventive medicine, and novel treatments. Complex diseases are caused by multiple genetic, environmental, and behavioral factors. If a disease has heterogeneous etiologies, then the detection of operable genes is difficult as one set of genes can be important for one etiology, but not another. Therefore, the identification of the genetic determinants of complex diseases is difficult. Endophenotype is an intermediate phenotype that combined genetic factors associated with a disease to reduce genetic heterogeneity [1]. This approach assumes that complex diseases can be described by sets of simple and measurable disease characteristics, with each characteristic representing a basic biological phenomenon. In the literature, synonyms for endophenotype include intermediate phenotype, biological marker, and sub-clinical trait, although each term has slightly different implications [2]–[5]. The endophenotype approach may be useful in exploring different pathways leading to the onset of a complex disorder. For example, patients with the same diagnosis may differ greatly in the number and severity of symptoms, suggesting heterogeneity in the causal pathways [6]–[8]. Therefore, the creation of more homogeneous subgroups of patients based on their endophenotypes may facilitate our understanding of the involved biological processes.

The identification of disease subtypes is important because homogeneous groups likely reflect stronger clinical, pathological, and genetic coherence, and this may facilitate the understanding of the mechanisms underlying a disease. The molecular heterogeneity of a complex disease may suggest the existence of molecular subtypes [9], [10]. Genomic tools such as DNA microarrays hold great potential for the deciphering of the molecular patterns of disease and the identification of new and improved clinical markers. Gene expression profiling has been applied extensively to studies on gene function, gene regulation, cellular processes, and disease subtypes. Many human genes show natural variation in expression levels [11], [12], which suggests that gene expression levels may be used to establish endophenotypes to identify genes that confer disease susceptibility [13]–[15].

In general, gene expression datasets contain thousands of genes derived from a relatively small number of samples. The gene expression data can be represented by a matrix 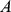 (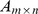) of 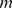 transcripts in 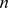 samples. As such, standard statistical methods are not appropriate for analyzing gene expression data. Unsupervised clustering methods represent an alternative approach for exploring molecular dissimilarities among patients. To date, several methods have been applied for dimension reduction such as principal component analysis (PCA) [16], singular value decomposition [17], and independent component analysis [18]. These methods capture overall gene behaviors that cluster genes based on global similarities in their expression data [19]. Recently, Lee and Seung [20] proposed non-negative matrix factorization (NMF), a matrix factorization method, 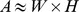, where the elements of 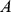, 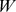, and 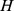 are all non-negative. NMF imposes non-negative constraints to detect local gene behaviors, in contrast with the approaches used by other linear representation clustering methods. NMF differs from PCA and singular value decomposition by enforcing the constraint that the two factors W and H must be non-negative, i.e. all elements must be equal to or greater than zero, and the factorization of matrices is generally non-unique. NMF has been applied to microarray data, protein sequence data, and data from neuroscience studies [21]–[23].

NMF creates a small number of gene subspaces from all of the genes in a genome and summarizes the sample gene expression patterns in each of the gene subspaces [24]. These gene expression patterns are then used to cluster samples into distinct tumor types and subtypes. NMF is superior to both hierarchical clustering and self-organizing mapping in subtype discovery. A previous study has compared the performance of dimension reduction using NMF and PCA with several widely studied, related cancer microarray datasets and has shown that NMF outperformed PCA in reducing data dimensionality [25].

In the present study, with the advantages of higher clustering accuracy and superior dimension reduction, we investigated an endophenotype-identification analytical procedure that first applies NMF to explore the potential molecular dissimilarities of a complex disease based on high-throughput microarray data and then uses the adjusted rand index (ARI) [26]. ARI is a measure of the similarity of two clustering groups of a data set. In our study, ARI was applied to select informative transcripts for each molecular subtype. Both NMF and PCA are methods that reduce the dimension linearly and aim to find a small set of transcripts that describe the underlying information contained in 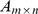 with a lower dimension, 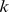. We also used a simulation to compare the efficiency of NMF and PCA with k-means analysis (PCA-K) for endophenotype construction. Finally, we used a publicly available dataset derived from patients with late-onset Alzheimer’s disease (LOAD) [27] to evaluate the feasibility of our proposed procedure.

## Materials and Methods

### Endophenotype-identification Analytical Procedure: Molecular Subtype Construction via NMF

**Figure 1:**
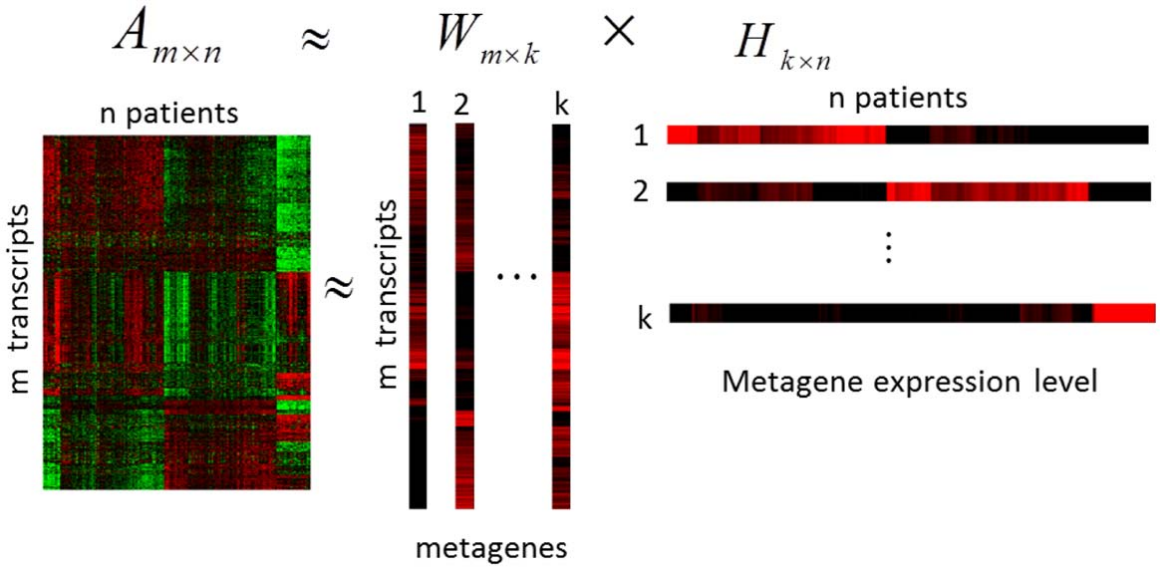
Schematic representation of factorization in NMF method. NMF decomposes matrix 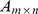 into 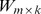 and 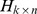. The columns 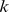 of matrix 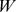 are called metagenes and the entry 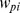 of matrix 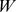 is the coefficient of transcript 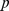 in metagene 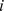 (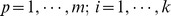). The entry 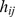 of matrix 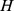 represents the expression level of metagene 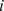 in sample 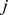 (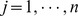).

NMF is an algorithm based on decomposition by parts that can reduce the dimension of expression data from thousands of genes to a handful of gene sets [24]. When applying NMF to a matrix 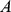, the matrix 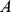 can be factored into two matrices 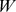 and 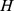 with 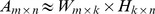, where the columns 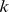 of matrix 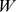 are called metagenes, as defined by Brunet et al. [24]. Figure 1 showed the factorization structure [24]. In this study, the entry 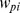 of matrix 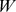 is the coefficient of transcript 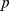 in metagene 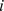. The entry 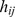 represents the expression level of metagene 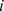 in sample 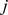. Each column of matrix 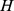 represents the metagene expression level of the corresponding patient sample. After applying NMF with an appropriate value of 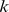, a 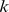-dimension metagene expression level will be generated for each sample, 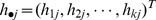 for sample 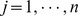. In this study, the standard NMF factorization with an algorithm adopted from Lee and Seung [13] was used to produce two non-negative matrices, 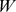 and 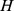. The factorization process was begun by randomly initializing matrices 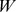
and 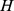 and then iteratively updating them to minimize a divergence function 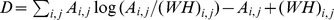 for metagene 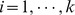 and sample 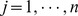. To apply NMF to construct endophenotypes, the entry 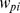 of matrix 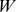 was regarded as the importance value of transcript 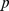 for 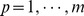 in metagene 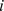 for 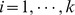. Transcripts with large 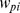 values contributed more toward the metagene when 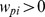 for any 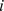 value. Note that the dimension was reduced from 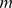 transcripts to 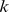 metagenes. Regarding 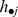, sample 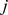 was placed in cluster 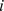 if 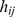 was the largest entry in 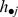, as suggested by Brunet et al. [24].

One key issue in NMF is choosing an appropriate 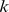 such that the samples decompose into 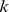 “meaningful” clusters. To evaluate how many clusters were appropriate, a widely accepted criterion, the cophenetic correlation coefficient 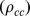, which is based on a consensus matrix, was used to determine cluster number [24], [28]. The 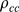 is a measure to evaluate the stability of the clusters from NMF. It is computed by the Pearson correlation of two distance matrices. The first matrix is the consensus matrix which measures the distance between samples. The second distance matrix is made by the linkage used in the reordering of the consensus matrix [24]. The entries of the consensus matrix range from 0 to 1 and reflect the probability that samples 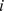 and 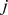 cluster together. Based on the idea of consensus clustering, 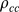 range from 0 to 1. A large 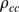 indicates high clustering stability; therefore, the number of clusters 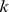 with the largest 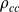 was chosen. To visualize the sample clustering stability associated with a given cluster number 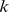, average linkage hierarchical clustering were used to reorder the columns and rows of the consensus matrix [24]. For this study, the R software (http://www.r-project.rg) [76] package “NMF” [29] was used to perform NMF.

### Endophenotype-identification Analytical Procedure: Informative Transcript Selection for Biological Interpretation of Metagenes

Most entries 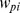 of matrix 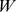 were close to zero, meaning that metagene was determined by a relatively small number of transcripts with large importance values. In addition, redundant transcripts in 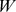 complicated the biological interpretation of metagenes and identification of molecular subtypes. To exclude redundant transcripts, we determined which transcripts were most informative for each metagene by ranking each column of matrix 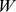. The transcript selection process was performed as follows. First, for each 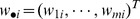, the entry 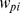 in 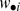 for 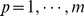 was ranked in descending order. Given a certain value 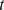, the top 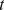 transcripts were selected for each metagene 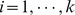. Then, another round of NMF was carried out, based solely on non-redundant 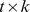 transcripts selected from 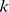 metagenes.

To choose an appropriate 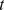, the process was repeated 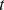 times for each metagene. ARI is a frequently used cluster validation measurement that verifies the agreement between two partitions [26]. In the present study, let 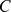 be the set that contained non-redundant 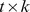 transcripts and 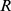 be the set that contained 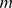 transcripts. ARI was used to assess the agreement of the molecular subtypes between set 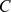 and set 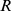. ARI values range from 0 to 1. Higher index values indicate more agreement between sets. In this study, the minimum 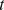 among those with ARI ≥0.95 was selected as the optimal informative transcripts set. The selected transcripts were then used for pathway analyses.

MetaCore™ (GeneGo, Inc., St. Joseph, MI, USA) is a web-based computational platform designed for systems biology. It provides tools for gene list enrichment analysis, multi-experiment comparison, interactome analysis, and biological network analysis [30], [31]. The biological characteristics of each molecular subtype were determined using MetaCore pathway analysis. The top 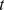 transcripts that overlapped among 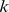 metagenes were excluded in the MetaCore pathway enrichment analysis.

### PCA-K

PCA is a common method that is used to provide a simple overview of complex structure using a small number of principal components (PCs) to reduce high-dimension data. The PCs are linear combinations of original transcripts that explain the variation in the data and are orthogonal to each other. In gene expression analysis, the PCs have been referred to as metagenes, as introduced by Ma et al. [32]. Briefly, to apply PCA to transcription data, the data matrix 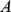 with 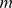 transcripts and 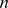 samples was decomposed by singular value decomposition to 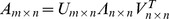, where 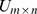 was a score matrix, 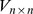 was a loading matrix, and 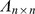 was a matrix containing the so-called singular values. Matrices 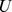 and 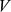 were orthogonal. To find potential molecular subtypes, the score matrix was used for clustering with the standard k-means clustering method and the squared Euclidean distance measure [25].

### Simulation Studies

For comparing the performance between NMF and PCA-K in constructing endophenotype, we considered informative genes and non-informative genes based on the idea proposed by Fogel P. et al [33] in order to mimic real microarray data. In the simulation studies, we considered three different molecular subtypes, T1, T2, and T3, each with an equal sample size of 80. The non-informative genes represented those that had no contribution in distinguishing molecular subtypes. First, for simulating non-informative genes, the gene expression level was described by the model
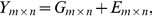
(1)where matrix 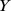 (the gene expression data) had size 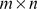 with rows representing genes and columns representing samples, matrix 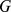 was the log_2_-transformed mean of the overall gene expression level using log_2_ of 100 [33], matrix 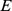 was the multivariate random variable simulated from a multivariate normal distribution with a mean vector of zero. We assumed that all genes in a molecular subtype were correlated at the same level. Hence, to simultaneously simulate multiple molecular subtypes, the covariance matrix was considered as a diagonal partition consisting of the correlation matrix for each molecular subtype. The variance of each gene was set to be 0.3, and the correlation level between genes was set to moderate dependence ranging from 0.4 to 0.6 [34]. In addition, the proportion of non-informative genes, 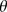, was assumed at 70%, 80%, and 90% [33].

Recent works [35]–[37] has demonstrated that co-regulated genes are expected to rise and fall together in expression levels. The genetic variation can be used to identify groups of genes that might share a common background. Correlated variation in groups of genes may result in common phenotypes [38]. To add the variation in gene expression to our simulation, we presumed that the difference in gene expression level between molecular subtypes was resulted from the variants in the DNA sequences (i.e., single-nucleotide polymorphisms [SNPs]) [39]. Hence, for informative genes, we used a linear model to link the relationship between SNP data and gene expression as proposed by many eQTL studies such as Stranger et al. [40] and Chen et al. [41]. We assumed a one-to-one correlation between a SNP and a gene expression level, that is, the expression level from microarray is related to SNP allele frequency. Therefore, a group of SNP markers were selected by fixing the difference of minor allele frequencies (MAF) (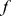) to predict a subtype. To include informative genes, equation 1 was rewritten as follows:
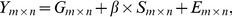
(2)where matrix 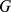 and matrix 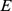 were the same as in equation 1. Matrix 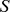 (the genotype data) was generated from a multinomial distribution with consideration of various minor allele frequency (MAF) differences among subtypes. To simulate informative genes, 160 subtype-specific overexpressed genes were generated for each of the molecular subtypes. To simulate overexpressed genes in T1, the MAF was set to 0.1+ 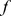 for subtype T1, and the MAF was set to 0.1 for subtypes T2 and T3, where 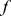 was the MAF difference between T1 and the other subtypes (T2 and T3). Similarly, the process for generating overexpressed genes was repeated for other subtypes. An additive genetic model was used, which coded copies of the minor allele as 0, 1, or 2. The coefficient 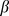 represented the magnitude of the SNP effect on gene expression level. Finally, matrix 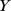 in equation 2 was applied to both NMF and PCA-K.

To evaluate the performance of NMF and PCA-K in constructing endophenotypes, an accuracy measure, *purity* [42], was used. Assuming 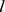 is the true number of clusters, after applying a clustering method (e.g. NMF and PCA-K), 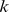 clusters are obtained. *Purity* is given by the following equation [42]:
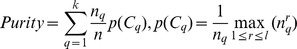
where 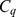 is a particular cluster of size 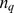 from a clustering method, 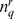 is the number of samples in cluster 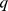 that belongs to the original cluster *r 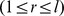
* and 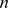 is the total number of samples. Hence, *Purity* indicates the proportion of samples in cluster 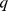 being clustered correctly in cluster 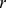. The larger the value of *purity* is, the better the clustering performance. All simulation scenarios were run 1000 times. The average purity measure was calculated for each run.

### Application to LOAD Dataset

To demonstrate the feasibility of our analytic procedure in an endophenotype study, we used a publicly available dataset derived from patients with LOAD [27]. The dataset is freely available at http://labs.med.miami.edu/myers/. LOAD is a common complex neurodegenerative disorder that occurs in people over the age of 65 years. The dataset contained data on 176 patients with a diagnosis of LOAD and on 188 patients who were neurologically normal. The cRNA data contained 8,650 transcripts that were obtained using the Illumina Human Reseq-8 Expression Bead Chip following standard operating procedures as described in Webster et al. [27]. The DNA data contained 372,084 autosomal SNPs that were obtained using the Affymetrix Gene Chip Human Mapping 500 K Array set.

Before evaluating our proposed procedure, we preprocessed the LOAD dataset. First, transcripts were eliminated when expression values for a given transcript were missing for >30% of the subjects. This resulted in the removal of 338 transcripts. For the remaining 8,312 transcripts, log_2_-transformed values were used to reduce outlier impact and ensure normality. Many of the genes on the array were not expressed, were expressed at low levels, or were expressed at a level with no biological significance. To further reduce the effects of irrelevant or noisy variables, we selected transcripts with mean expression values in the upper 70^th^ percentile and with variances in the upper 50^th^ percentile, as suggested by Langfelder et al. [43]. The remaining 1,116 transcripts met all filtering criteria. Missing data from these 1,116 transcripts were replaced by the mean value of the transcripts across patients. Therefore, further analyses were constructed without any missing data.

Rare alleles are more likely to result in spurious finding due to a higher relatedness between individuals sharing rare alleles [44]. In addition, loci with a low MAF (<10%) have significantly lower power to detect weak genotypic risk [45], [46]. Therefore, to avoid spurious findings resulting from rare genotypes, SNP markers with low MAFs were excluded; specifically, we adopted the criteria of removing SNPs with MAF <10% [47], which resulted in a total of remaining 283,475 SNP markers for further analysis. For incomplete genotype data, “beagle” software [48] was used for imputation. Due to the exploratory nature of our study, a loose threshold (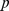-value <10^−4^) was used as the significance criterion in differential expression and metagene expression QTL analyses.

## Results

### Simulation Study

**Figure 2:**
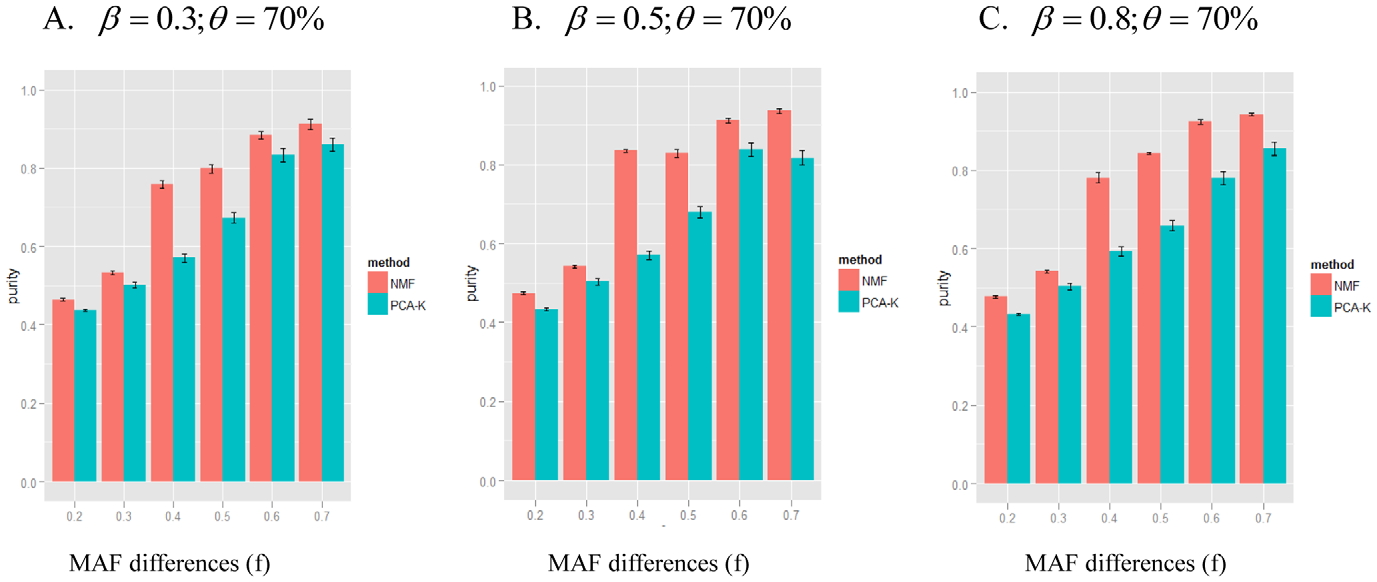
Simulation results of NMF and PCA-K for various 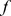 and 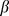 at 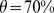. The simulations for a range of MAF differences (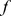) and the magnitude of SNP effect (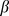) under the proportion of non-informative genes 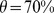. The x-axis represents the MAF differences. The y-axis represents the average purity given by NMF (red) and PCA-K (blue). The average purity of each method was shown as mean
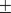
standard error. A-C indicated 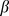 = 0.3 (A), 0.5 (B), and 0.8 (C), respectively.

**Figure 3:**
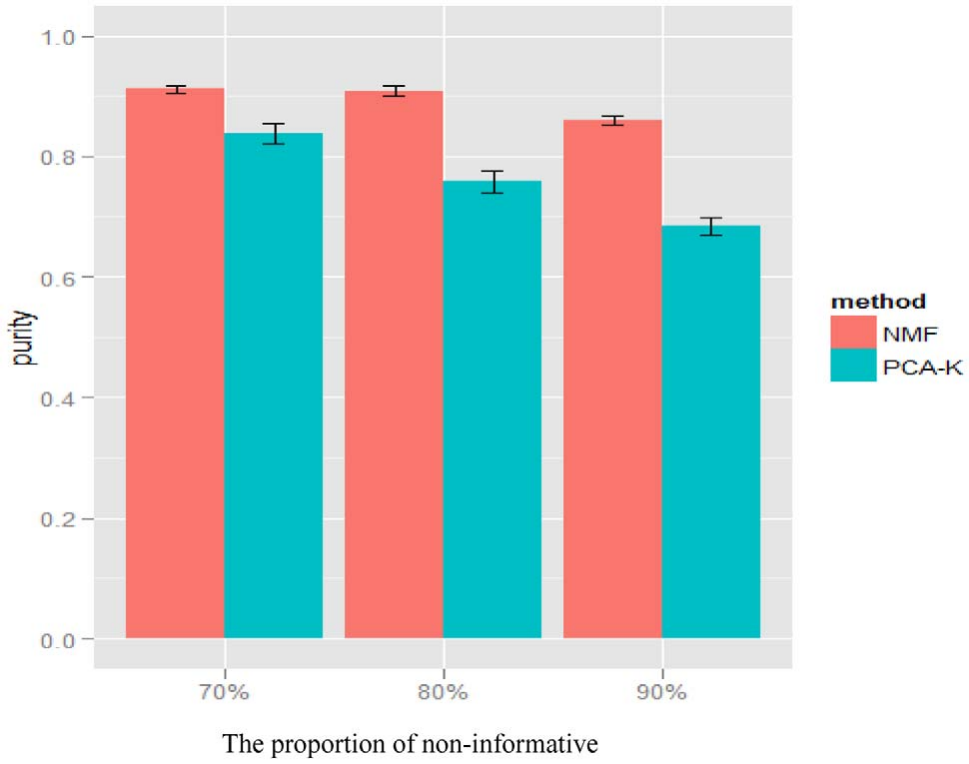
Simulation results of NMF and PCA-K for various proportions of non-informative genes 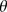. The simulations for a range of proportions of non-informative genes (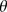) under MAF difference 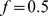 and magnitude of SNP effect 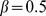. The x-axis represents the proportions of non-informative genes. The y-axis represents the average purity given by NMF (red) and PCA-K (blue). The average purity of each method was shown as mean
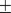
standard error.

Because the results for the simulated scenarios were similar, Figure 2 demonstrates the average purity results for NMF and PCA-K with various values for 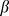, 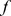
and 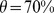. Figure S1 shows results for 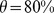 and 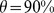 with various values for 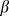 and 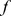. As shown in Figure 2, the NMF method performed better than the PCA-K method under different scenarios. When the MAF difference, 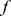, was <0.4, the average purity of the NMF and PCA-K methods was close to 0.6 under different 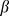 values. This indicated that the classification using the NMF and PCA-K methods was not satisfactory when the dissimilarity among subtypes was not clear. With increasing values of 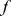, the average purity of NMF and PCA-K methods was higher under different 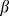 values. The average purity of the NMF method increased dramatically at 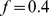, whereas the average purity of the PCA-K method increased sharply at 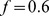. These results showed that NMF had a better sensitivity than PCA-K for detecting divergence among subtypes. Additionally, with different 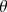 values and under 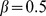 and 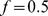, the average purity for NMF and PCA-K decreased slightly (Figure 3). This finding indicated that the impact of noise was not strong.

### Results for the LOAD Dataset: Molecular Subtypes Among Patients

**Figure 4:**
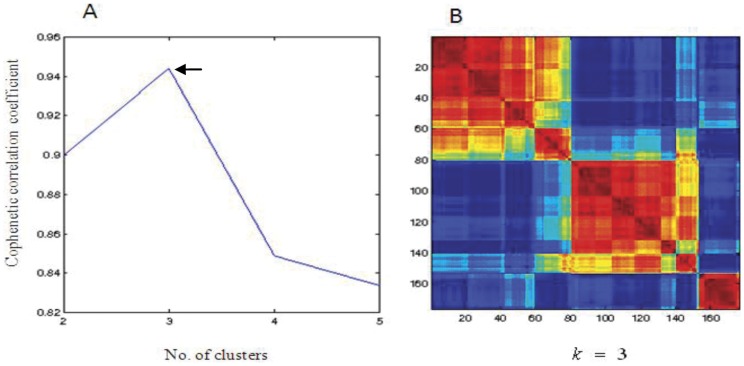
Unsupervised clustering using NMF. (A). Cophenetic correlation coefficients associated with different numbers of clusters 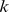. Y-axis is the cophenetic correlation coefficient (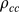); *x*-axis is the number of clusters. The arrow displays the 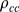 at 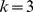. (B). Heat map of reordered consensus matrix for 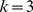. Dark blue indicates samples never assigned to the same cluster, and red indicates samples always assigned to the same cluster.

We applied NMF to the LOAD dataset containing 1,116 transcripts derived from 176 patients. A cophenetic correlation coefficient (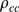) was calculated for each value of 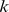. Figure 4A shows 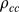 levels corresponding to 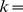
2 to 5. 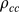 peaked at 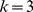 (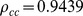) and fell off sharply as 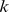 increased, indicating that 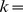
3 was the best fit for this dataset. Three clusters yielded the highest 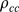 values, which reduced the initial dimensionality of the 1,116 transcripts to three metagenes and grouped the 176 patients into three molecular subtypes: MS1 (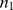 = 80), MS2 (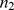 = 73), and MS3 (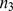 = 23) shown in Figure 4B.

Hence, NMF decomposed the 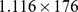 LOAD transcription data matrix into matrix 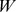 and matrix 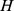. The non-negative matrix 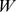 was 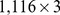, with each of the three columns representing a metagene. The non-negative matrix 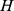 was 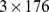, with each of the three rows representing the metagene expression levels for the corresponding sample. The three metagenes captured gene expression patterns specific to three different molecular subtypes of patients. The three metagene expression levels were named MGL1, MGL2, and MGL3, respectively.

**Figure 5:**
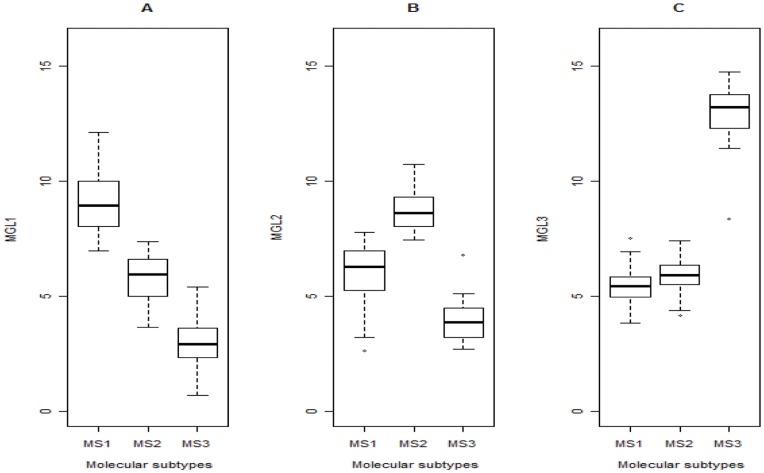
Association of metagene expression levels with molecular subtypes. Boxplots indicate metagene expression levels (MGL1–3) for each molecular subtype (MS1–3) in the LOAD samples. (A) MGL1 is the highest in MS1. (B) MGL2 is the highest in MS2. (C) MGL3 is the highest in MS3.

Devarajan [49] used metagene expression levels to determine which sample subtypes belonged to which specific metagenes. We therefore adopted the same approach. Figure 5 shows boxplots of the MGL1–3 values for each molecular subtype. Patients grouped in MS1 had higher MGL1 values as compared with patients grouped in MS2 or MS3 (Figure 5A). Patients grouped in MS2 had the highest MGL2 values (Figure 5B). The greatest differences were seen for MGL3, with patients grouped in MS3 having the highest MGL3 values (Figure 5C). In this way, MGL1, MGL2, and MGL3 represented distinct biological characteristics of molecular subtypes MS1, MS2, and MS3, respectively.

### Results for the LOAD Dataset: Informative Transcript Selection for Molecular Subtypes

The 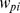 values corresponding to 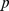 transcripts in the 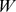 matrix were sorted in descending order within each metagene, 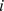 (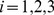). To identify the most relevant 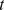 transcripts for each metagene, eight subsets were selected, i.e. 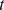 = 50, 100, 150, 200, 250, 300, 400, and 800, respectively. Non-redundant transcript subsets for the eight values of 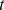 when 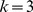 were listed in Table 1. The non-redundant transcripts represented the union of the top 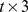 transcripts for the 3 metagenes. For example, when the top 50 transcripts (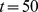) were chosen for each metagene (total 150 transcripts), 137 transcripts were the union of the 150 transcripts from 3 metagenes (Table 1). The transcript subset with 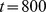 contained all 1,116 transcripts and thus was not considered further. NMF was then applied seven times to obtain new molecular subtypes for the seven remaining transcript subsets. Using a molecular subtype containing all 1,116 transcripts as the reference group, ARI values were calculated to evaluate molecular subtype agreement between transcript subsets, 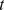, and the reference group. According to our criteria, 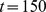 was the minimum 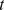 with an ARI value of >0.95 and therefore was selected (Table 1). In comparison with the reference group, only one patient was misclassified with the NMF method. Therefore, based on this analysis, the top 150 transcripts for metagenes 1, 2, and 3 were used to represent the biological characteristics of molecular subtypes MS1, MS2, and MS3, respectively. To interpret the biological characteristics of each molecular subtype, overlapped transcripts (i.e., those found in more than one metagene) were excluded to ensure uniqueness, and 123, 89, and 71 metagene-specific transcripts were identified for MS1, MS2, and MS3, respectively.

**Table 1:** Identification of the optimal transcript subset for molecular subtype representation.

No. of top transcripts t chosen for each metagene	Transcript subset^a^	Adjusted Rand Index (ARI)	No. of sample misclassification
50	137	0.94596	4
100	255	0.92589	5
150	366	0.9869	1
200	475	0.97929	2
250	583	0.97929	2
300	681	0.97929	2
400	846	0.97929	2
800	1116	1	0

a Number of transcripts that are union of the top t x 3 transcripts for 3 metagenes.

### Results for the LOAD Dataset: Enrichment Pathway Analysis via MetaCore

Furthermore, the metagene-specific transcripts were used to conduct a pathway analysis using the GeneGO pathway map from MetaCore. The pathways for each metagene-specific transcript with a significance of p-value <10^−3^ are shown in Table 2.

**Table 2:** Significant pathways for metagene-specific transcripts.

Metagene-specific transcripts	Pathways^a^	Nodes^b^	Gene ID^c^	pValue
Metagene 1	Development Gastrin in differentiation of the gastric muscosa	4/38	PRKCB1;CHGA	7.11E-05
	Cytoskeleton remodeling Neuroflaments^d^	3/25	NEFM;VIM;TUBA1B	4.19E-04
	Neurophysiological process Dopamine D2 receptor transactivation of PDGFR in CNS	3/26	GRIN1;PPKCB1;PPP2R2B	4.71E-04
Metagene 2	Immune response Function of MEF2 in T lymphocytes	4/50	PLCG1;PRKCZ;PPP3CB	3.39E-05
	Cytoskeleton remodeling Neurofilaments^d^	3/25	INA;STXBP1;TUBB	1.06E-04
	Neurophysiological process Role of CDK5 in presynaptic signaling	3/28	SH3GL2;STXBP1;SNAP25	1.50E-04
	Signal transduction cAMP signaling	3/38	PPP3CB;PRKCZ	3.76E-04
	Translation Insulin regulation of translation	3/42	EIF4A2;EIF4B;PRKCZ	5.06E-04
	Regulation of lipid metabolism Regulation of lipid metabolism by niacin andisoprenaline	3/45	PRKCZ;PPP3CB	6.21E-04
	Immune response NF-AT signaling and leukocyte interactions	3/46	PLCG1;PPP3CB	6.62E-04
	Innume response NFAT in immune response	3/51	PLCG1;NFAT5;PPP3CB	8.97E-04
Metagene 3	Development EPO-induced PI3K/AKT pathway and Ca(2+) influx	3/43	GAB2;HBB;HBA2	2.20E-04

Pathway enrichment analysis was conducted using metagene-specific transcripts. Significant biological pathways were detected by MeatCore at a significance level p-value <10^−3^.

Pathways are listed in order of significance, e.g., most significant pathway are presented at the top.

a Name of biological pathway selected by MetaCore.

b The number of metagene-specific transcripts associated with pathway/the number of all genes associated with pathway.

c Gene ID of metagene-specific transcripts associated with pathway.

d Different genes in "Cytoskeleton remodeling Neurofilaments" pathway were identified in metagene 1 and metagene 2.

### Enriched Pathways in Metagene1-specific Transcripts

In metagene1-specific transcripts, there were three significant enriched pathways. In the *Development Gastrin in differentiation of the gastric muscosa* pathway, *Gastrin* is a peptide hormone produced primarily by G cells, endocrine cells located in the gastric antrum. Transcription of the *Gastrin* gene gives rise to a 0.7 kb mRNA coding for a 101 amino acids precursor, known as Progastrin. Progastrin is processed to mature amidated *Gastrin* (*Gastrin 17*). *Gastrin 17* mediates its effects primarily through Cholecystokinin B receptor *(CCKBR*) [50], [51]. The *CCKBR* is a seven transmembrane G-protein coupled receptor (*GPCR*) that is expressed in the gastric fundus, parietal ECL and D cells. *GPCR* are involved in numerous key neurotransmitter systems in the brain that are disrupted in Alzheimer's disease (AD) [52]. The second enriched pathway was C*ytoskeleton remodeling Neurofilaments*. Cytoskeleton of most eukaryotic cells consists of three distinct, yet interconnected, filament systems: actin filaments, microtubules and intermediate filaments (IF). Neurofilaments are the principal intermediate filament type expressed by neurons. The third enriched pathway was *Neurophysiological process dopamine D2 receptor transactivation of PDGFR in central nervous system (CNS)*. Dopamine is a major transmitter and neuromodulator in the *CNS*. This transmitter mediates its signaling through *GPCRs*. Dysregulation of dopamine receptor activity account for neuropsychotic disorders such as Parkinson’s diseases, schizophrenia, and Alzheimer’s disease [53].

### Enriched Pathways in Metagene2-specific Transcripts

There were eight enriched pathways in metagene2-specific transcripts. We briefly described the top 3 significant enriched pathways. The first pathway was *Immune response function of MEF2 in T lymphocytes*. The transcription factor *Myocyte Enhancer Factor 2* (*MEF2*) is a family of muscle-enriched transcription factors that have an essential role in myogenesis. It has been implicated as playing a pivotal role in neuronal survival as well as in the development, differentiation, and plasticity of the *CNS* [54], [55]. The second pathway was *Cytoskeleton remodeling Neurofilaments*. In Table 2, we found that the metagene1-specific transcripts and metagene2-specific transcripts shared the same pathway. However, the genes in this pathway were different in metagene1-specific transcripts and metagene2-specific transcripts. This suggested that the genes in metagene1-specific transcripts had different process link to this pathway compared to the genes in metagene2-specific transcripts. The third pathway was *Neurophysiological process role of CDK5 in presynaptic signaling*. Cyclin-dependent kinase 5 (*CDK5*) is a member of the small serine/threomine cyclin-dependent kinase family with high activity in the central nervous system [56].

### Enriched Pathways in Metagene3-specific Transcripts

The significant enriched pathway in metagene3-specific transcripts was *Development EPO-induced PI3K/AKT pathway and Ca (2+) influx*. Erythropoietin (*EPO*) is a lineage-specific hematopoietic growth factor required for survival, proliferation and differentiation of committed erythroid progenitor cells [57], [58]. *EPO* has been shown to have multiple effects on neurons [59].

### Application of Molecular Subtypes in the LOAD Dataset: Differential Expression Analysis

To demonstrate the application of the molecular subtypes identified by our procedure, we compared discrepancies in differential expression analysis between patients with LOAD and normal subjects. We also conducted a differential expression analysis for patients with each molecular subtype and compared it to that for the normal subjects. A *t*-test was used to identify differentially expressed transcripts among the 1,116 transcripts, with significant thresholds of p-value <10^−4^. Our results found that 660, 887, 30, and 736 transcripts were differentially expressed for all patients and for patients grouped in MS1, MS2, and MS3, respectively, when compared with transcripts from normal subjects. Only 3 of the 660 differentially expressed transcripts identified for all patients were not included among those identified for patients grouped in the molecular subtypes. In comparison, it is worth noting that only 30 transcripts were significant for patients grouped in MS2, which suggests that MS2 might represent a normal-like subtype.

**Figure 6:**
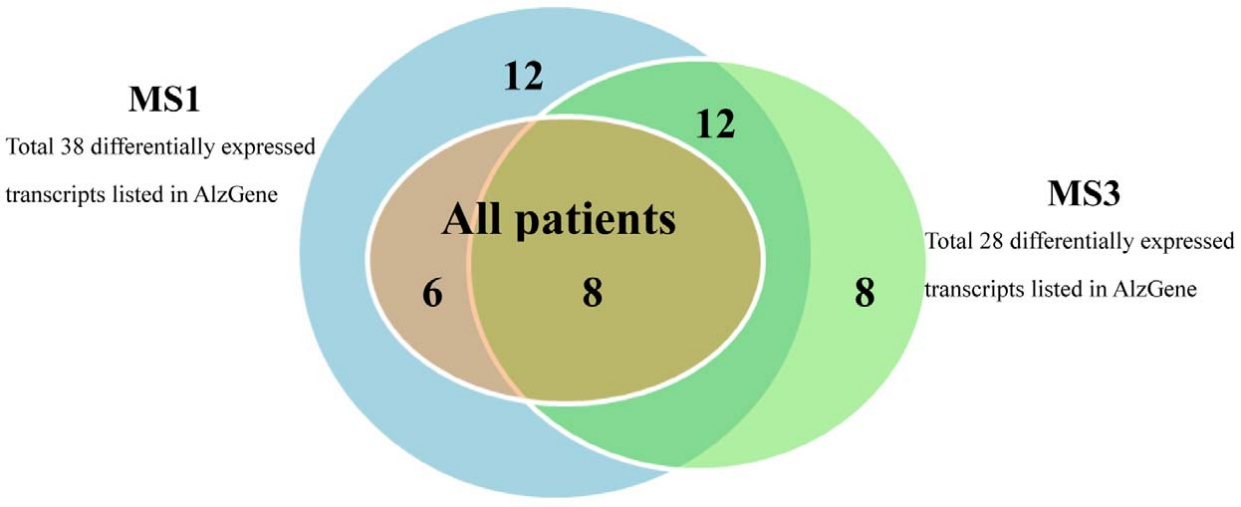
Venn diagram of differentially expressed transcripts listed in AlzGene. The Venn diagram showed the differential expressed transcripts in all patients, MS1, and MS3. These differentially expressed transcripts were listed in AlzGene database.

Furthermore, when comparing all patients with normal subjects, there were 660 differentially expressed transcripts. Among those transcripts, 14 were also listed in AlzGene database [60]. These 14 differentially expressed transcripts detected using all patients were also found from the results when comparing normal subjects with MS1 (Figure 6). In addition, 8 out of the 14 differentially expressed transcripts were intersected with differentially expressed transcripts identified when comparing normal subjects with MS3. We noted that there was no candidate susceptibility genes listed in AlzGene found as differentially expressed transcripts for MS2.

The gene encoding apolipoprotein E (*APOE*) has been identified as playing an important role in the development of Alzheimer’s disease [61]–[63]. In the differentially expressed analysis, we found that *APOE* gene was not significantly different (p-value = 0.3641) comparing all patients with normal subjects. When patients were grouped into three subtypes, the p-values of the differential expression analyses were 1.03×10^–11^, 0.00087, and 0.00182 for MS1, MS2, and MS3, respectively (comparing with normal subjects). At the significant threshold p<10^−4^ that we chose to use in this study, *APOE* gene was significant when comparing MS1 with normal subjects. A boxplot was drawn to show the expression level of *APOE* gene for 3 molecular subtypes, all patients and normal subjects. The median expression level of *APOE* gene was the highest in MS1 (Figure S3).

### The Validation of Molecular Subtypes in the LOAD Dataset

To validate the differential expression analysis derived from each molecular subtype in contrast to normal subjects, the changes of effect size were evaluated by Cohen’s *d* [64]. The results showed that the distribution of effect size in 1,116 transcripts for patients grouped in MS2 centered at zero and variation was small, thus, MS2 had little difference from normal subjects (Figure S2A). The distribution of effect size in 1,116 transcripts for patients grouped in MS1 and for patients grouped in MS3 had larger variation compare to all patients (Figure S2A). This showed that patients grouped by NMF enhanced the effect size in the differential expression analysis.

To examine whether the existence of heterogeneous subtypes among patients was random or not, a permutation strategy was used as suggested by Allison et al. [65]. In the permutation analysis, we randomly regrouped patients into three molecular subtypes. The differential expression analysis was then carried out in each regrouped subtype compared to normal subjects, and the effect size was recorded. The empirical distribution of effect size for the randomly grouped patients with 1,116 transcripts was estimated. We combined the effect size across 1,000 permutations according to average quantiles of empirical distribution. The distribution of effect size for the randomly grouped patients was very similar to that observed for all patients, regardless of the proportion of patients that were grouped together (Figure S2B). Therefore, these findings indicated that the definition of molecular subtypes in the LOAD dataset presented in this study is meaningful and can improve the efficiency of the identification of differentially expressed transcripts.

### Metagene Expression Quantitative Trait Locus (QTL) Mapping

To explore the genetic variants that are responsible for the molecular subtypes identified by differential transcript profiling, the genotype data were used to identify associations between genetic variants (i.e., SNPs) and metagene expression levels. Associations between SNP markers and each of the three MGLs were tested using a linear regression model in which individual MGLs were each regressed on individual SNP markers, and the significance level was set at a threshold 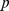-value of 10^–4^. The results showed that 11, 25, and 31 SNP markers were associated with MGL1, MGL2, and MGL3, respectively, and were named MGL1-QTL, MGL2-QTL, and MGL3-QTL, respectively.

To explore the additive property of the effects of these SNP markers on molecular subtype identification, the frequencies of up-regulated alleles in each MGL-QTL were calculated for MS1–3. The up-regulated allele was the one that had a positive coefficient in the regression model; in other words, the up-regulated allele enhanced the metagene expression level. The up-regulated alleles were called u-alleles. This analysis was intended to help understand the possible relationship that the SNPs regulated metagene expression levels for a specific molecular subtype. We calculated the mean allele frequency of u-alleles for each MGL-QTL. The mean allele frequency of u-alleles of 11 MGL1-QTL was higher in MS1 (0.6658) than in MS2 (0.5924) and MS3 (0.4895). Similarly, the mean allele frequency of u-alleles related to the 25 MGL2-QTL was higher in MS2 (0.4925) in comparison with MS1 (0.3797) and MS3 (0.3248); the mean allele frequency of u-alleles of 31 MGL3-QTL had higher frequency in MS3 (0.3050) than in MS1 (0.1370) and MS2 (0.1494). The results showed that the molecular subtypes with its respective MGL had higher frequencies of u-alleles in the LOAD data.

## Discussion

Patients with a common disease can display molecular heterogeneity [66]. These molecular differences often are important because they can reflect the biological processes that underlie pathogenic diversity [67]. Molecular markers such as transcripts or protein levels can be used to identify molecular dissimilarities among patients. Identification of these specific disease subtypes could dramatically improve our understanding of disease pathology.

In our simulation study, the magnitude of the SNP effect on gene expression level (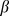), the MAF difference among subtypes (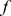), and the proportion of non-informative genes (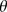) were considered to evaluate the efficiency of endophenotype construction using the NMF and PCA-K methods. Our findings demonstrated that the magnitude of the effect of MAF difference (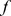) was larger than the magnitude of the SNP effect on gene expression level (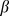). This finding might result from our assumption that SNPs with differences in allele frequencies varied significantly between molecular subtypes. The difference might result from different patterns of expression levels among different molecular subtypes. In previous cancer microarray studies, NMF was used to examine the efficiency of detection of potential disease subgroups [68], [69]. Our simulations provide an alternative insight for the detection of molecular subtypes.

In our simulation study, the variations of gene expression levels were controlled only by the difference of MAF of some specific SNP markers among subtypes. In reality, other factors such as heterogeneity of genetic effect between different subtypes or different direction of effect between subtypes may influence gene expression levels. When these effects are considered in our method, the diversity of gene expression level will increase. According to a previous study [49], NMF has good performance in class discovery when diversity of groups is high. Therefore, it is expected that NMF will still be useful in constructing endophenotypes.

In this study, we assumed that all genes in a molecular subtype were correlated at the same level in simulation study. To check this assumption, we further examined the variation of correlation for LOAD dataset. The average correlation with metagenes for each molecular subtype was calculated. The average correlation ± standard error were 0.4427±0.003, 0.2312±0.0028, and 0.6369±0.0031 for MS1, MS2, and MS3, respectively. The results showed that the assumption seems to be acceptable for the simulation study.

One of the advantages of NMF is that it produces two non-negative matrices that provide a parts-based local representation of the data. In comparison with NMF, PCA produces both positive and negative entries in metagenes (i.e., PCs) that can cancel each other out, at least in part, and this creates difficulties in the capturing of local data characteristics [49]. In addition, entries associated with NMF are interpreted readily as the relative contribution of genes and are well grounded in physical reality [26]. Another advantage of NMF is that it can simultaneously cluster genes and patients. In this study, NMF was used to reduce data from thousands of transcripts into a small number of metagenes, 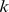. This simplification provided a summary of patient gene expression levels from corresponding metagenes and was used to extract informative patterns from complex data. For application in complex disease, we applied NMF to the LOAD dataset and successfully explored three potential molecular subtypes, MS1, MS2, and MS3.

There have been several improvements to the standard NMF method. In our present study, we further applied non-smooth NMF (nsNMF) [19], an extended method by making use of non-smoothness constraints, to simulations at a few different values of MAF difference among subtypes (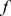) and magnitude of the SNP effect on gene expression level (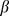) based on a fixed proportion of non-informative genes (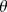 = 70%). Overall speaking, the average purity of for nsNMF was better than standard NMF (Figure S4). The results indicated that nsNMF method refined standard NMF method in endophenotype construction.

In addition to NMF, we proposed to incorporate the process of informative transcript selection in the identification of molecular subtypes. Here we used ARI to measure agreement between molecular subtypes to ensure that the utilization of the top selected transcripts,t, for each metagene captured the same molecular subtypes as when all transcripts, 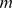, were used. Consequently, we anticipated that the correlations between metagene expression obtained from the selected and the entire set of transcripts would be high. For the LOAD dataset, the correlation coefficients for the metagene expression levels between the top 150 transcripts and the original 1,116 transcripts were 0.9844, 0.9840, and 0.9772 for MGL1, MGL2, and MGL3, respectively. As a result, the proposed process could help to exclude redundant transcripts and to maintain a similar molecular pattern as that observed with the original m transcripts.

In order to find the biological characteristics for each molecular subtype, the metagene-specific transcripts that overlapped among 3 metagenes were excluded to decrease the noise for finding the specific characteristics for each molecular subtype in pathway analysis. The removal of these genes may lose potential information of showing linkages between pathways.

To evaluate the predictive power of three metagene expressions in molecular subtypes, a multinomial logistic regression using three metagene expression levels with three molecular subtypes was constructed. The predictive power was almost 98%. The result showed that these metagene expression levels predict molecular subtypes well.

In addition to log_2_ transformation to preprocess the transcripts profiling, we also used arcsinh transformation. To examine whether transformation methods changed clustering, we then applied NMF method to the data set and utilized ARI index to compare the clustering between results obtained from the two transformation methods. The ARI value was 0.9259 which indicated that the results for the two transformation methods were similar. Therefore, it seems that the impact of using different transformation methods to the clustering results is mild.

In our real data, we found that it was feasible to construct endophenotype using data mining tools with genomic data in LOAD study. To utilize the most information in LOAD study, microarray data was used to construct endophenotypes and SNP data was used to explore the correlation between endophenotypes and SNP markers for representing the underlying etiology of Alzheimer’s disease. However, for some phenotypes, microarray data may not fully represent the underlying genetic etiology. For example, microarray expression level from lymphocyte drawn from peripheral blood may not well represent patients’ blood pressure [70]. Due to differential spatial and temporal expression patterns, some genes are only expressed in specific tissues [71]. One of the limitations of NMF is that it can only be applied to continuous data. Other clustering methods e.g. random forest that can handle discrete data can be considered if only SNP data is available.

We have considered several thresholds of p-value for association analysis between metagene gene expression level (MGL) and SNP marker. Since this was an exploratory study, we chose to use a loose threshold p-value <10^−4^. Therefore, proper multiple testing was not fully considered.

When examining significant MGL-QTLs, there was no overlapping SNP marker among molecular subtypes. This result might be due to the exclusion of overlapping SNP markers before conducting QTL analysis. Therefore, it is still possible that there is some heterogeneity of genetic effects between different subtypes.

In addition, we found that numerous candidate susceptibility genes in the AlzGene database could not be reproduced using data from all patients. This finding may be due to the dilution effect contributed by the normal-like subtype, MS2. These results showed that the use of the entire patient dataset may fail to detect potentially important susceptibility genes that are specific to molecular subtypes of patients, as identified using our proposed procedure.

In our study, we utilized a permutation study to examine whether the existence of molecular subtypes among patients was random or not. The results showed that the identified molecular subtypes were apparent in LOAD patients. Moreover, we also applied another popular clustering method, PCA-K, to LOAD dataset and found 3 molecular subtypes. Results showed that the clustering pattern of PCA-K was similar to those of NMF with ARI  = 0.98 and both of them could identify a normal-like subtype. These results indicated the existence of heterogeneous subtypes in LOAD patients.

In pathway analysis, the candidate pathways obtained from our study have not been identified for Alzheimer’s disease related studies, however, we found that the three enriched pathways in metagene1-specific transcripts were related to neurotransmitter and neuronal system [72], [73]. The enriched pathways in metagene2-specific transcripts were related to neuronal system and immune system [56], [74] and the enriched pathway in metagene3-specific transcripts reported effect on learning and memory [75]. The results showed that each molecular subtype with its respective metagene has specific characteristics. This finding may help us to understand the pathophysiology of Alzheimer’s disease.

Therefore, a consideration of the molecular differences among patients is recommended to identify the genetic causes of a complex disease. However, if one tests multiple genetic subtypes, one should adjust the statistical testing accordingly. Moreover, a potential disadvantage focusing on molecular subtypes is that statistical power may be compromised when large data set is reduced to create homogeneous subgroups. Hence, further study regarding power reduction in endophenotype using smaller data set with possible stronger genetic effect should be considered. Carefully chosen endophenotypes may reveal alternative pathophysiological disease processes. Collectively, our findings suggest that this proposed approach is an effective method for constructing complex disease endophenotypes.

## Supporting Information

Figure S1
**Results of NMF and PCA with k-means for simulation.** The simulations for a range of magnitude of SNP effect (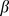) and proportions of non-informative genes (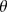). The x-axis represented the MAF differences 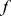. The y-axis represented the average purity given by NMF (red) and PCA-K (blue). The average purity of each method was shown as mean
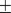
standard error.(TIF)

Figure S2 **The validation results of molecular subtypes with LOAD data.** (A) Patients grouped by NMF: the plot showed the distribution of effect size in 1116 transcripts for all patients (black) and molecular subtypes (MS1 = red, MS2 = green, and MS3 = blue). (B) All patients randomly grouped: the plot showed the empirical distribution of effect size in randomly grouped patients by average quantile across 1000 times of permutations (entire patients = black, MS1 = red, MS2 = green and MS3 = blue).
(TIF)

Figure S3 **The gene expression level of** ***APOE*** **gene for all patients, control subjects and molecular subtypes.** Boxplots indicate the gene expression level of *APOE* gene for all patients, control subjects and each molecular subtype (MS1–3).
(TIF)

Figure S4
**Simulation results of NMF and nsNMF for various** 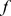 **and** 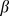 **at 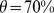.** The simulations for a range of MAF differences (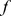) and magnitude of SNP effect (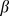) under the proportion of non-informative genes 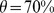. The x-axis represents the MAF differences. The y-axis represents the average purity given by NMF (red) and PCA-K (blue). A-C indicated 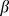 = 0.3 (A), 0.5 (B), and 0.8 (C), respectively. The average purity of each method was shown as mean
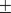
standard error.(TIF)
