# Supplementary figures and images for: Constructing Endophenotypes of Complex Diseases Using Non-Negative Matrix Factorization and Adjusted Rand Index
Source: PLoS One. 2012 Jul 16;7(7):e40996. doi: 10.1371/journal.pone.0040996 (PMC3397992; doi:10.1371/journal.pone.0040996)

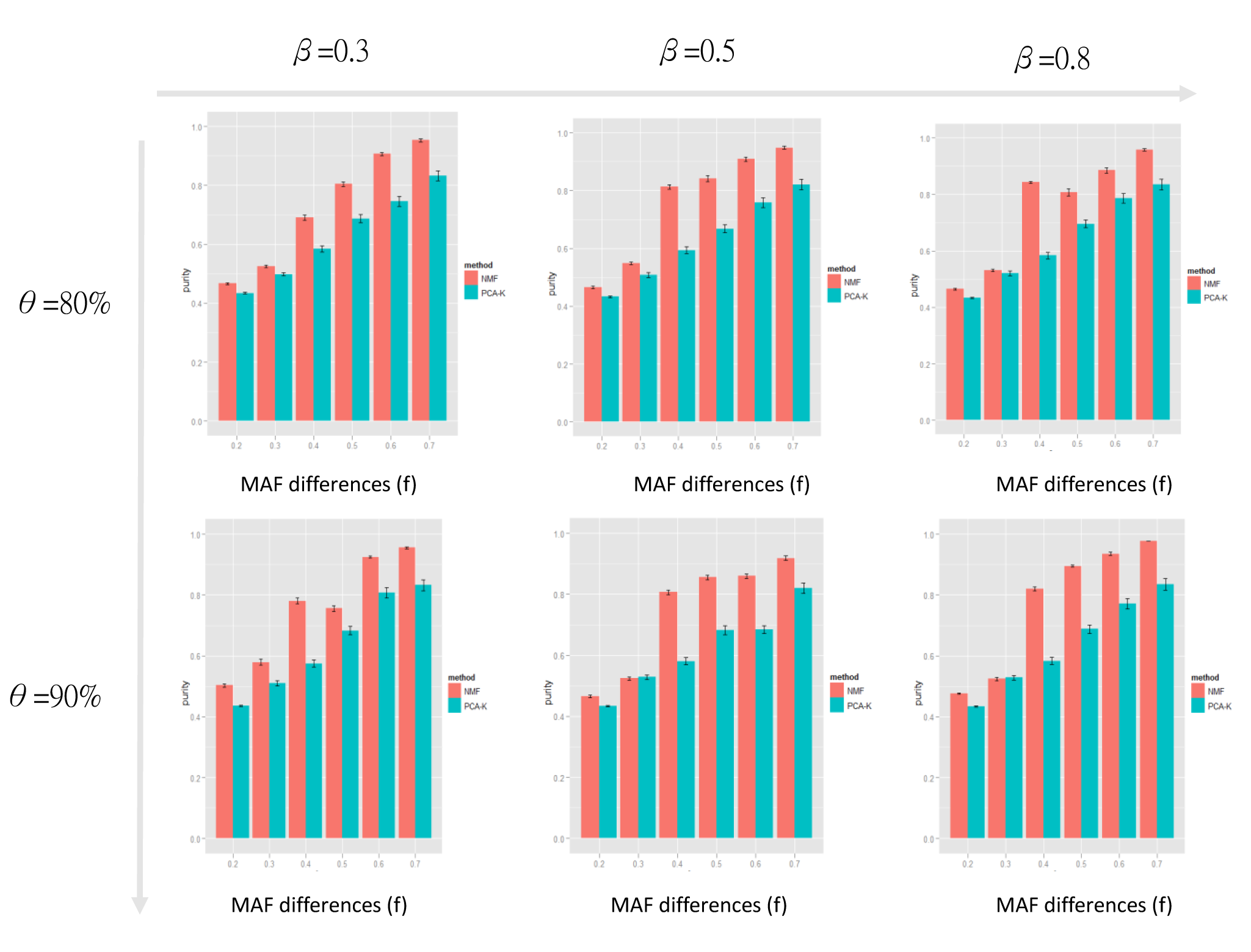

Supplement: Figure S1 — Results of NMF and PCA with k-means for simulation. The simulations for a range of magnitude of SNP effect () and proportions of non-informative genes (). The x-axis represented the MAF differences . The y-axis represented the average purity given by NMF (red) and PCA-K (blue). The average purity of each method was shown as meanstandard error. (TIF) [file pone.0040996.s001.tif]

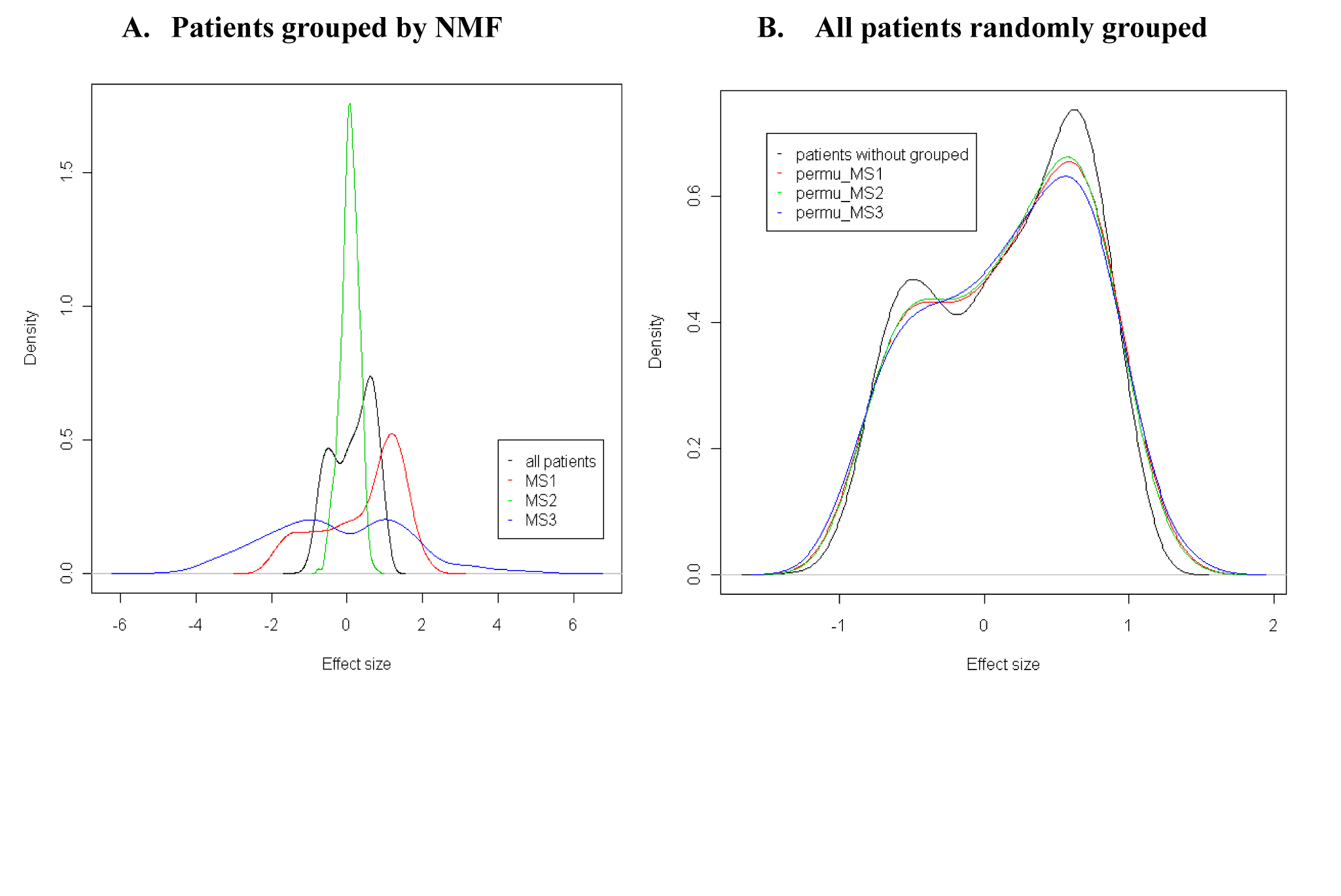

Supplement: Figure S2 — The validation results of molecular subtypes with LOAD data. (A) Patients grouped by NMF: the plot showed the distribution of effect size in 1116 transcripts for all patients (black) and molecular subtypes (MS1 = red, MS2 = green, and MS3 = blue). (B) All patients randomly grouped: the plot showed the empirical distribution of effect size in randomly grouped patients by average quantile across 1000 times of permutations (entire patients = black, MS1 = red, MS2 = green and MS3 = blue). (TIF) [file pone.0040996.s002.tif]

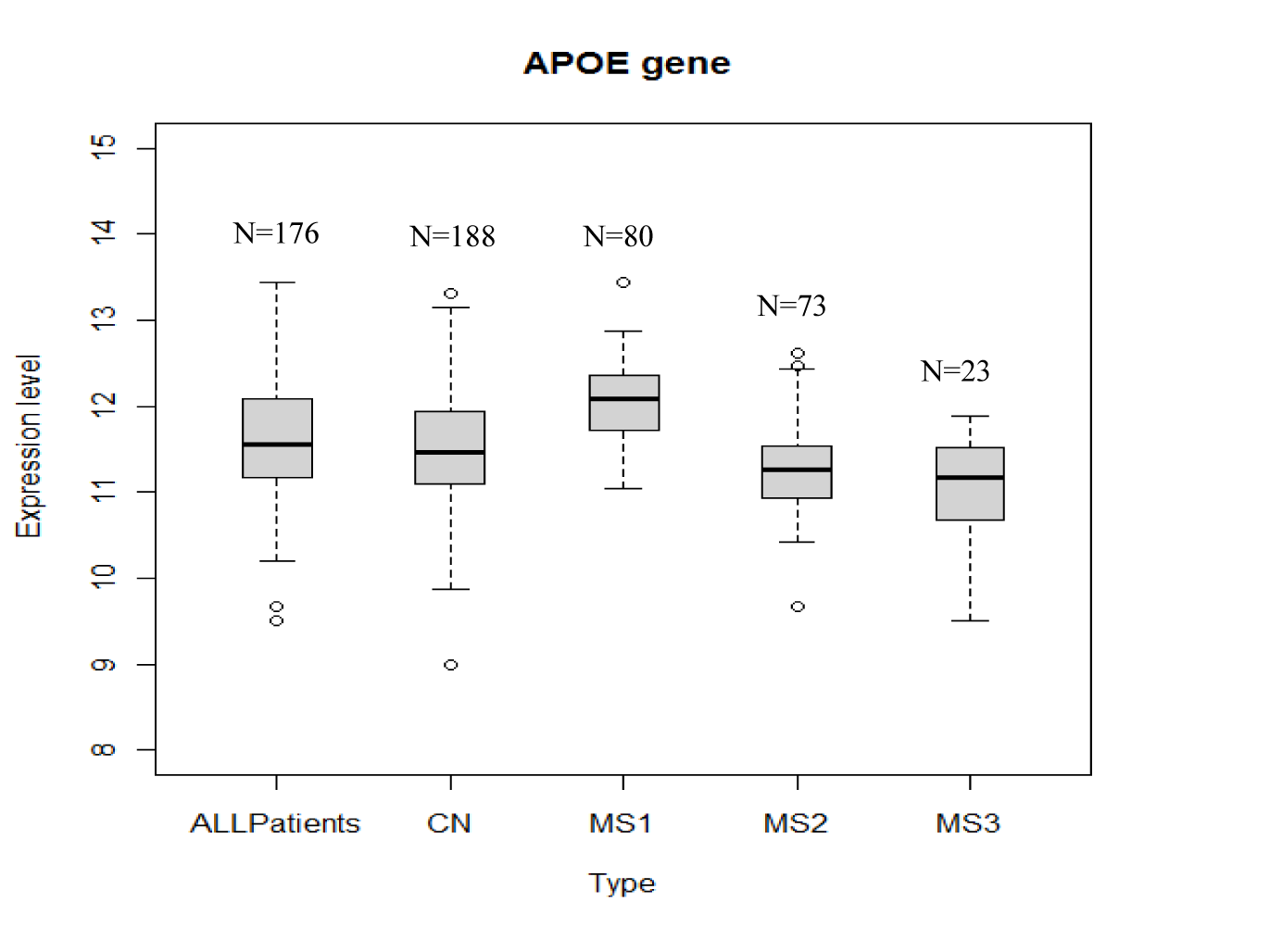

Supplement: Figure S3 — The gene expression level of APOE gene for all patients, control subjects and molecular subtypes. Boxplots indicate the gene expression level of APOE gene for all patients, control subjects and each molecular subtype (MS1–3). (TIF) [file pone.0040996.s003.tif]

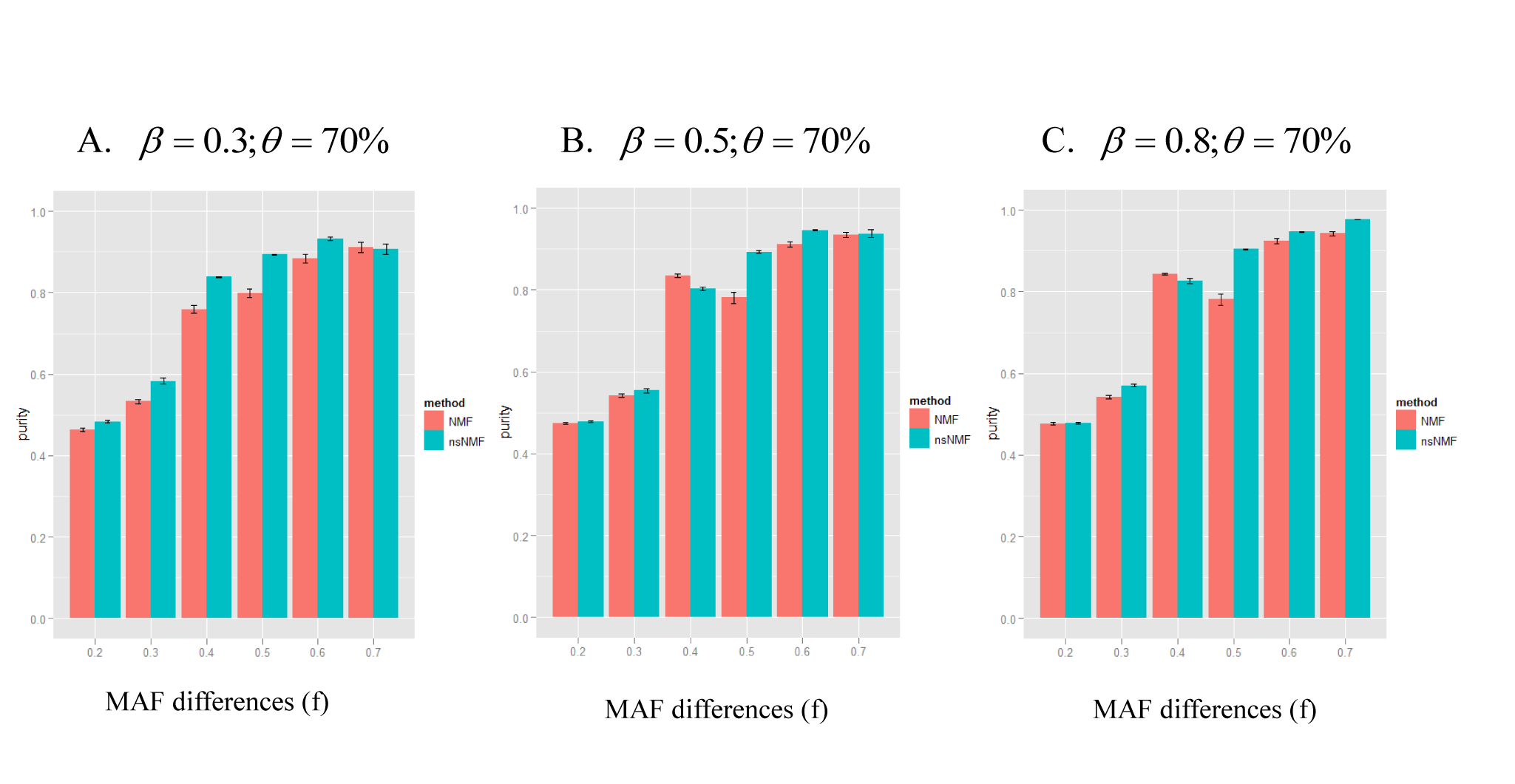

Supplement: Figure S4 — Simulation results of NMF and nsNMF for various and at . The simulations for a range of MAF differences () and magnitude of SNP effect () under the proportion of non-informative genes . The x-axis represents the MAF differences. The y-axis represents the average purity given by NMF (red) and PCA-K (blue). A-C indicated = 0.3 (A), 0.5 (B), and 0.8 (C), respectively. The average purity of each method was shown as meanstandard error. (TIF) [file pone.0040996.s004.tif]
